# Validation of high-throughput, semiquantitative solid phase SARS coronavirus-2 serology assays in serum and dried blood spot matrices

**DOI:** 10.4155/bio-2021-0065

**Published:** 2021-06-11

**Authors:** Leo Maritz, Nicholas J Woudberg, Amber C Bennett, Andreia Soares, Florian Lapierre, Justin Devine, Matti Kimberg, Patrick J Bouic

**Affiliations:** ^1^Synexa Life Sciences, 4 Kunene Circle, Montague Gardens, Cape Town, 7441, South Africa; ^2^Trajan Scientific & Medical, 7 Argent Place, Ringwood, Victoria, 3134, Australia; ^3^Division of Medical Microbiology, Faculty of Medicine & Health Sciences, University of Stellenbosch, Cape Town, 7505, South Africa

**Keywords:** COVID-19, dried blood spot, ELISA, neutralizing antibodies, SARS-CoV-2, serology

## Abstract

**Aim:** Serological assays for the detection of anti-SARS coronavirus-2 (SARS-CoV-2) antibodies are essential to the response to the global pandemic. A ligand binding-based serological assay was validated for the semiquantitative detection of IgG, IgM, IgA and neutralizing antibodies (nAb) against SARS-CoV-2 in serum. **Results:** The assay demonstrated high levels of diagnostic specificity and sensitivity (85–99% for all analytes). Serum IgG, IgM, IgA and nAb correlated positively (R^2^ = 0.937, R^2^ = 0.839, R^2^ = 0.939 and R^2^ = 0.501, p < 0.001, respectively) with those measured in dried blood spot samples collected using the hemaPEN^®^ microsampling device (Trajan Scientific and Medical, Victoria, Australia). *In vitro* SARS-CoV-2 pseudotype neutralization correlated positively with the solid phase nAb signals in convalescent donors (R^2^ = 0.458, p < 0.05). **Conclusion:** The assay is applicable in efficacy studies, infection monitoring and postmarketing surveillance following vaccine rollout.

Coronavirus disease 2019 (COVID-19) is an infectious viral pneumonia caused by the most recently discovered zoonotic coronavirus (SARS coronavirus-2 [SARS-CoV-2]). As of May 2021, the disease remains a proponent of a global pandemic while vaccine rollouts aim to combat its spread [1].

A coronavirus is a positive-sense, enveloped ssRNA virus. Several structural glycoproteins are expressed on or in the lipid envelope of SARS-CoV-2, namely the membrane (M), envelope (E) and spike (S) proteins. The spike protein is among the most immunogenic viral structural peptides and plays a pivotal role in host cell entry. The SARS-CoV and SARS-CoV-2 species of coronaviruses specifically target the angiotensin-converting enzyme 2 (ACE2) receptor, expressed on type II pneumocytes and endothelial, myocardial, and gut mucosa cells [2]. Anti-SARS-CoV-2 neutralizing antibodies (nAbs) targeting the S1 subunit of the spike protein are central to many current and potential therapies, as they play an important role in interrupting entry and viral proliferation [3].

The initial seroconversion of IgM antibodies to SARS-CoV-2 can occur as little as 3 days post illness onset (PIO) and peak at 14–21 days PIO. IgG and IgA seroconversion occurs 4+ days PIO, peaking after 14–17 days [4]. IgA isotypes are less prevalent in serum, but are the most abundant antibodies in mucosal tissues such as the lungs and respiratory tract. Serology assays that screen for the presence and relative abundance of IgG, IgM and IgA antibody isotypes may assist in monitoring recovery from SARS-CoV-2 infection [5]. This can also provide insight into the development of virus-targeted adaptive immunity, and crucially, be employed for postmarketing surveillance following administration of vaccines [4].

Serological tests typically require on-site collection of blood specimens, performed by a healthcare practitioner through phlebotomy or finger prick. The need for in-person sample collection limits access to testing for individuals due to distance from testing sites, disability, inconvenience or social distancing. Furthermore, the venepuncture process requires the services of qualified personnel, thereby limiting service capacity and increasing testing costs [6]. To negate these factors, and to decrease the risks involved for both patients and staff through aggregation at sample collection centers and clinics, decentralized sample collection is a favorable alternative.

While many sample collection protocols are amenable to decentralized testing, the small sample volume requirements for analysis and the stability of antibodies at ambient temperatures on filter paper – dried blood spots (DBS) – may also provide a viable decentralized solution to conventional sample collection for serological assays [7–9]. While traditional DBS techniques have been around for decades, their use has been limited due to factors such as sampling volume variability, cross-contamination and hematocrit-based biases [10]. Modern collection devices such as the hemaPEN^®^ microsampling device (Trajan Scientific and Medical) have been developed to address these limitations while leveraging its native advantages, including low sampling volume, stable antibody storage before analysis and decentralized quality blood sampling.

As the disease landscape continues to rapidly evolve, including the rise of new mutant strains [11] and the massive rollout of vaccines, the need to monitor disease progression, humoral immunity and vaccine effectiveness is paramount. Since serology assays serve as well-suited candidates to effectively detect humoral immunity in patients, the aim of the current study was to validate solid phase assays, according to US FDA and European Medicines Agency regulatory guidelines, for the semiquantitative detection of anti-SARS-CoV-2 antibodies in serum samples. Parameters for the validation included assay performance and repeatability, diagnostic sensitivity (DSn) and diagnostic specificity (DSp), cross-reactivity, isotype stability, and assay robustness. In addition, the assays were compared with *in vitro* SARS-CoV-2 neutralization and to samples collected using the hemaPEN^®^ microsampling device from patients.

## Patients & methods

### Subjects

All volunteers provided written informed consent. Volunteers included were PCR-confirmed positive and recovered (convalescent) COVID-19 patients, as well as healthy controls. At the time of sample collection, all subjects were asymptomatic. There were no exclusion or inclusion criteria. This study was approved by the Human Ethics Committee of the University of Stellenbosch (N19/09/2020). Additional samples were donated by a local biorepository for further analysis. These samples included a panel of subjects who were exposed to other bacterial or viral pathogens (excluding SARS-CoV-2), and contained antibodies against such pathogens (e.g., hepatitis B, human immunodeficiency virus [HIV] and syphilis). We also considered samples from volunteers with autoimmune conditions including rheumatoid arthritis (RA), psoriasis, chronic inflammatory demyelinating polyneuropathy and myasthenia gravis (MG).

### Sample preparation

Venous blood was drawn into serum-separating tube (SST) sample collection tubes and centrifuged. Serum was aliquoted and stored at -70°C before analysis, according to standard laboratory procedures. DBS samples were collected according to manufacturer’s instructions. Briefly, a lancet was used to perform a finger prick and the first drop of blood was removed. Four 2.74-μl blood samples were simultaneously collected and stored on four 3.5-mm paper disks (Whatman 903) from the finger prick site using a hemaPEN^®^ device. Within 7 days of collection, DBS samples were eluted into LowCross Buffer^®^ (Candor Bioscience; Cat: 100500, Allgäu, Germany) by shaking (350 ± 50 r.p.m.) overnight at 2–8°C and following elution, stored at -70°C before analysis.

Positive controls (PCs) were prepared from pooled prescreened convalescent subjects who were positive for all four anti-SARS-CoV-2 analytes. On the day of analysis, individual frozen aliquots of 100% pooled positive subject serum were thawed and diluted with pooled negative serum to the respective control levels: high PCs (90%, HPC), medium PCs (60%, MPC) and low PCs (30%, LPC).

### SARS-CoV-2 serology assay

The 384-well clear microplates (Greiner, Cat: 781061, MO, USA) were coated overnight at 2–8°C with 0.250-μg/ml COVID-19 S1 protein His Tag (ACRO Biosystems, Cat: S1N-C52H3, DE, USA). Plates were washed with phosphate-buffered saline-Tween (0.05%) before blocking at ambient temperature (20–25°C) with SuperBlockTM blocking buffer (Thermo Fisher Scientific; Cat: 37515, MA, USA) for at least 90 min. Samples were diluted with LowCross Buffer^®^ (Cat: 100500) to 4% serum for IgM, IgA and nAb analysis, and 0.4% serum for IgG analysis. DBS samples collected using hemaPEN^®^ microsamplers (Cat: 498100011) were eluted in LowCross Buffer^®^ and diluted to 1% matrix for IgM, IgA and nAb analysis, and 0.4% matrix for IgG analysis. Subject samples and quality control samples were loaded and incubated for 1 h at ambient temperature with shaking at 400 r.p.m.

Detection reagents used (prepared at 0.250 μg/ml) were horse-radish peroxidase (HRP-conjugated) goat anti-human IgM (Invitrogen; Cat: 31415), goat anti-human IgG (Invitrogen; Cat: 31410), goat anti-human IgA and human ACE2 (ACRO Biosystems; Cat: AC2-H52H8, DE, USA). Human ACE2 was used for the competitive detection of anti-SARS-CoV-2 nAbs. Detection reagents were incubated at ambient temperature for 1 h with shaking at 400 r.p.m. Moreover, 3,3′,5,5′-tetramethylbenzidine (TMB) substrate was added, and plates incubated at ambient temperature with shaking at 400 r.p.m. (10 min incubation for IgG detection and 30 min incubation for IgA, IgM and nAb detection) before 0.172 M HCl was added to stop the reaction. Absorbance was measured at 450 nm.

### Assay cut-point determination

The type of assay cut point (ACP) was determined independently for each analyte to achieve optimal specificity and sensitivity. Two methods of calculating the ACP were applied, namely: receiver operating characteristic (ROC) analysis for IgG, IgM and IgA isotypes or the implementation of a dynamic ACP by evaluating the between-assay signal means and variances generated from a panel of SARS-CoV-2 naive samples for nAb analysis.

Briefly, ROC analysis of the ACP determination for each analyte was based on the visual inspection of the transformed signal distribution of SARS-CoV-2 naive and confirmed convalescent samples (including PC samples) in a dedicated dataset. The ACP dataset for each analyte (using the first set of positive samples) was compiled from nine independent assays. A ROC curve area of r ≥ 0.900 was deemed acceptable for use in ACP determination [12]. The signal data used in ACP determination for each analyte was normalized to plate-specific HPC mean. Therefore, for all data generated for IgG, IgM and IgA was normalized to the plate-specific HPC mean.

For nAb analysis, the signal mean and SD from a panel of at least ten naive samples were used to determine a plate-specific dynamic ACP [13].

### DSn & DSp

DSn and DSp for each analyte were determined based on the test results from samples with known infection status (i.e., SARS-CoV-2 naive and confirmed SARS-CoV-2 convalescent subjects). DSn and DSp of each analyte were determined from the results for SARS-CoV-2 negative (naive) controls, confirmed convalescent subject samples as well as the SARS-CoV-2 prepared PCs analyzed during ACP determination.

DSn and DSp were calculated using the following formulae:

$$Diagnostic\;Sensitivity\;(DSn)=\frac{TP}{TP+FN}$$

$$Diagnostic\;Specificity\;(DSp)=\frac{TN}{TN+FP}$$

Where TP is the number of true positive samples identified, TN is the number of true negative samples identified, FP is the number of false positives (FPs) identified and FN is the number of false negatives identified.

### Assay performance

Assay performance was measured by examining intra-assay (within-run) and interassay (between-run) variability. Assays were conducted with at least six replicates of freshly prepared samples at each PC level, frozen PC samples, and a panel of subject samples consisting of at least 15 PCR-confirmed SARS-CoV-2 convalescent individuals and at least 40 SARS-CoV-2 naive individuals. Eight interassay (between-run) assessments were performed over multiple days by more than one analyst.

### Cross-reactivity

Nonspecific antibody cross-reactivity was assessed from a panel of human serum samples derived from SARS-CoV-2 negative (naive) donors with autoimmune diseases (RA, psoriasis, chronic inflammatory demyelinating polyneuropathy and myasthenia gravis) and those who had been exposed to other viruses (excluding SARS-CoV-2). The human samples in the cross-reactive panel were analyzed as a single replicate, and detection of all four antibody targets (IgA, IgG, IgM and nAb) was determined. The immunogenic response obtained from SARS-CoV-2 seronegative samples in the cross-reactive panel was assessed against the response of the three PC levels (HPC, MPC and LPC), loaded in duplicate, as well as the ACP.

### Assay robustness & isotype stability

Sensitivity to small alterations in assay conditions was assessed for each analyte. This assessment included two assays wherein small alterations were made in the incubation temperature, duration and shaking speed applied during the method.

The on-bench stability assessment served to optimize the conditions under which clinical samples are to be thawed, and to determine the stability period from sample thaw to analysis under various conditions (e.g., at ambient temperature and 2–8°C). In addition, short-term sample stability was assessed to determine the integrity of thawed samples stored at 2–8°C for 24 h.

### *In vitro* pseudotyped SARS-CoV-2 neutralization assay

293T-hsACE2 cells (Integral Molecular; Cat: C-HA101, PA, USA) were cultured in Dulbecco’s modified Eagle medium (DMEM) (Gibco; Cat: 10-017-CVR, MA, USA) supplemented with 10% foetal bovine serum (FBS) (Thermo Fisher Scientific, Cat: 16140-071, MA, USA), 10 mM 4-(2-hydroxyethyl)-1-piperazineethanesulfonic acid) (HEPES) (Gibco, Cat: 15630-080, MA, USA) and 1% penicillin-streptomycin (Sigma-Aldrich; P4333, MO, USA) at 37°C with 5% CO_2_ and grown to 60–80% confluency. Cells were harvested using 0.05% trypsin-EDTA solution (Corning; Cat: 25-052-CI, NY, USA), centrifuged at 200 × *g* for 5 min, resuspended in cell culture media (10% FBS, 10 mM HEPES, 1% penicillin/streptomycin in DMEM) and counted prior to coculture. Serum samples from COVID-19 positive donors were thawed at ambient temperature and heat inactivated for 30 min at 56°C prior to preparing twofold serial dilutions (ranging from 1:5 to 1:80) in DMEM. Diluted serum was incubated in 96-well plates with equal volumes of pseudotyped SARS-CoV-2 green fluorescent protein (GFP) reporter viral particles (RVP) (Wuhan-Hu-1 strain; Cat: RVP-701G-10) for 1 h at 37°C before addition of 4 × 10^5^ 293T-hsACE2 cells. After 72 h of incubation at 37°C, cells were washed with BD Pharmingen™ Stain Buffer (BSA) (BD Biosciences; Cat: 554657), fixed in BD™ Stabilizing Fixative (BD Biosciences, Cat: 339860, CA, USA) and acquired on a BD FACSCanto II flow cytometer. Neutralization curves were normalized to no antibody controls (293T-hsACE2 cells cultured with RVP alone).

### Correlation to hemaPEN^®^ DBS samples

Assays which included serum controls at all three levels, serum and hemaPEN^®^ equivalent DBS samples for convalescent and naive individuals were performed using the standard serology assay at the specified matrix percentages.

### Statistical analysis

Assay acceptance criteria required all HPC, MPC and at least 50% of LPC replicates for all isotypes to test positive relative to the ACP. The CV between PC replicates of the same level should be ≤25% for all four analytes. Spearman correlation coefficients were determined for mean signals for all isotypes analyzed in serum and DBS sample matrices for equivalent samples. Additionally, Spearman qualitative correlation matrices (not assuming Gaussian distribution) for all isotypes analyzed in serum and DBS sample matrices for equivalent samples were determined. Similarly, with Spearman correlation, *in vitro* SARS-CoV-2 neutralization was correlated with serum matrix signals for all isotypes for equivalent samples.

## Results & discussion

In this study, we validated serum-based serology assays for the semiquantitative detection of anti-SARS-CoV-2 antibodies. The ligand binding assay (LBA) format allowed for inexpensive, rapid separate detection of IgG, IgM and IgA isotypes as well as SARS-CoV-2 nAbs. The assays demonstrated little intra and interday variability; high DSn and DSp including low levels of nonspecific antibody cross reactivity with other viruses and in individuals with chronic inflammation. Serum signals were strongly associated with signals generated from equivalent DBS matrix collected using the hemaPEN^®^ microsampling device, although sensitivity in DBS samples was lower than serum, which required adjustment in matrix percentages. Finally, our assays positively correlated with *in vitro* measurement of SARS-CoV-2 viral neutralization. These assays are therefore appropriate for postmarketing surveillance following vaccine rollout, monitoring of immune response in infected individuals as well as in vaccine efficacy trials. In situations where venous blood sampling is not feasible, safe or appropriate microsampling devices such as hemaPEN^®^ facilitate the blood sampling procedure while retaining the analysis using the validated assay.

Serological testing during the COVID-19 pandemic is a critical tool in managing the response to the disease. The detection of antibodies can determine past exposure of both symptomatic and asymptomatic patients. This, in combination with routine PCR testing, will be important in delineating disease severity and progression [14]. In addition, convalescent serum still presents a potential avenue for treatment; thus, the identification of individuals with high antibody levels remains essential.

The most common SARS-CoV-2 serological testing approach (utilized in our assays), is a focus on the S1 spike protein [4,15]. Other immunological targets include the receptor binding domain and nucleocapsid; however, low cross-reactivity with other coronavirus proteins make the S1 subunit an attractive target for serological assay builds [4]. Importantly, the immune response to SARS-CoV-2 infection is multifaceted, implicating multiple antibody isotypes. Our assays, which include three antibody isotypes as well as nAbs, cover this spectrum of immunological response. Inclusion of multiple antibody isotypes also mitigates the potential for reduced specificity [16].

**Figure 1.**
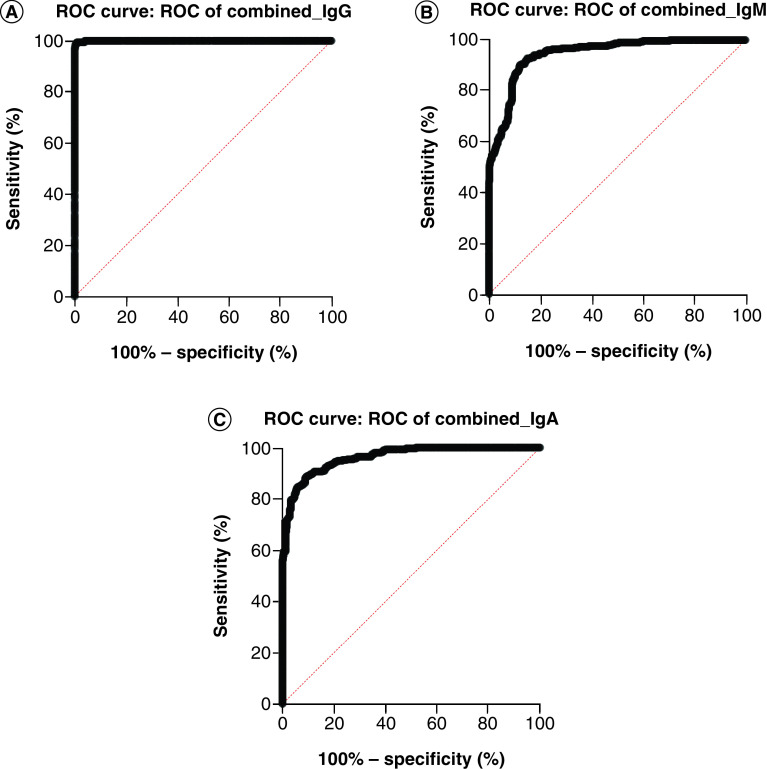
Receiver operating characteristic for IgG (A), IgM (B) & IgA (C) analytes. Transformed signal distribution of SARS coronavirus-2 naive and confirmed convalescent samples (including positive control samples) was included in the dataset across nine independent assays. A ROC curve area of r ≥ 0.900 was deemed acceptable for use in assay cut point determination. ROC: Receiver operating characteristic.

ROC curve analysis for IgG, IgM and IgA analytes is presented in Figure 1A–C. Data were constructed from nine independent assays and each included a total of 58 SARS-CoV-2 naive individuals, 21 confirmed convalescent individuals as well as PC samples at HPC, MPC and LPC levels. An area under the ROC curve of 0.9997 was attained for IgG with an ACP of 43.660 (% of mean HPC) (Figure 1A), 0.9460 was attained for IgM with an ACP of 45.010 (% of mean HPC) (Figure 1B) and 0.9622 was attained for IgA with an ACP of 40.200 (% of mean HPC) (Figure 1C). A ROC curve area of r <0.900 was determined for nAb, and therefore a dynamic ACP, based on differences in signal means and variances, was selected as an alternative approach to ACP determination.

Using the same datasets included for ACP determination, DSp and DSn were calculated for each analyte by applying the established ACPs. The results are summarized in Table 1. In terms of DSp, which relates to how a test successfully differentiates between true positive and FP samples, the results suggest a favorable DSp (>95% DSp) for IgG, IgA and nAb. DSn was determined using the same positive samples (i.e., confirmed convalescent and PC samples) for all four analytes based on the assumption that all analytes would be detected in every positive sample. IgG displayed the highest sensitivity (>99%), which compares well to automated IgG-only or total (IgG, IgM and IgA) serological assays [17,18]. Reduced sensitivity for IgA and IgM analytes was observed when measuring a panel of positive and naive samples. This was similarly observed by [17,19] wherein sensitivity was improved in symptomatic patients. The pentameric structure of IgM, along with a lower affinity for viral proteins compared with IgG, increases the likelihood of the detection of FPs [20]. Additionally, due to the time course of IgM seroconversion, the PIO time will greatly impact analyte levels in donor serum [21,22]. While our assays did display lowest specificity for IgM (90.5%) as well as a lower cross-reactivity specificity (85.7%) compared with other analytes, this level of specificity still compares well to similar assays [21].

**Table 1.** DsP and sensitivity for IgG, IgM, IgA and nAb analytes.

Parameter	Analyte ID			
	IgG	IgM	IgA	nAb
ACP type	ROC	ROC	ROC	Dynamic ACP
ACP value	43.660	45.010	40.200	n/a
Total negative samples	438	421	426	397
Total positive samples	307	308	311	271
False positives	4	44	15	18
FP ratio	0.009	0.105	0.035	0.045
False negatives	1	43	63	48
FN ratio	0.003	0.140	0.203	0.177
Specificity (DSp)	99.1	90.5	96.5	95.5
Sensitivity (DSn)	99.7	87.7	83.2	85.0

ACP: Assay cut point; DSn: Diagnostic sensitivity; DSp: Diagnostic specificity; FN: False negative; FP: False positive; n/a: Not Applicable; nAb: Neutralizing antibody; ROC: Receiver operating characteristic.

Since not all convalescent samples are positive for all antibody isotypes, additional DSn values were calculated exclusively from PC samples. Table 2 indicates that DSn for IgG remains unchanged (DSn >99%) while DSn for IgM, IgA (DSn >95%) and nAb (DSn >87%) were improved.

**Table 2.** Diagnostic sensitivity for IgG, IgM, IgA and nAb analytes.

Parameter	Analyte ID			
	IgG	IgM	IgA	nAb
ACP type	ROC	ROC	ROC	Dynamic ACP
ACP value	43.660	45.010	40.200	n/a
Total positive samples	162	161	160	143
FP	1	6	4	20
FP ratio	0.006	0.037	0.025	0.140
DSn	99.4	96.4	97.6	87.7

ACP: Assay cut point; DSn: Diagnostic sensitivity; FP: False positive; n/a: Not Applicable; nAb: Neutralizing antibody; ROC: Receiver operating characteristic.

The within-run performance of the PC samples was assessed using the data from a single run for each analyte, which included six replicates of each control at all levels (Table 3). All replicates of each PC level tested positive for IgG, IgM and IgA with the %CV ranging from 1.0–3.1%, 4.1–12.0% and 3.1–5.5%, respectively. All replicates of the HPC, MPC and four of the six LPC replicates tested positive for nAb with the %CV ranging from 5.2–8.0%.

**Table 3.** Within-run performance of positive control samples.

Statistic	Analyte, assay cut point											
	IgG, 43.660			IgM, 45.010			IgA, 35.657			nAb, 1.627		
	HPC	MPC	LPC	HPC	MPC	LPC	HPC	MPC	LPC	HPC	MPC	LPC
Mean (%/signal)	100.000	95.690	62.790	100.000	85.181	49.416	100.000	82.015	45.232	1.291	1.394	1.562
SD	0.975	1.559	1.965	9.477	10.231	2.043	3.076	3.006	2.482	0.067	0.084	0.126
%CV	1.0	1.6	3.1	9.5	12.0	4.1	3.1	3.7	5.5	5.2	6.1	8.0

HPC: High PC; LPC: Low PC; MPC: Medium PC; nAb: Neutralizing antibody; PC: Positive control.

The cumulative statistics for interassay repeatability of all four analytes is summarized in Table 4. The between-run performance of the nAb assays was assessed using signal data normalized to the plate-specific reagent blank sample. The findings from the between-run performance assessment suggest that satisfactory assay repeatability was obtained, as each analyte maintained a between-run covariance of <20% for all PC levels across the relevant assays.

**Table 4.** Between-run performance of positive control samples.

Statistic	Analyte ID											
	IgG			IgM			IgA			nAb		
	HPC	MPC	LPC	HPC	MPC	LPC	HPC	MPC	LPC	HPC	MPC	LPC
Mean (%)	100.000	96.721	67.022	100.000	84.163	52.429	100.000	81.025	44.690	66.402	74.996	85.680
SD	3.103	4.741	9.119	4.437	4.861	8.876	3.594	4.758	3.682	10.579	8.786	7.737
%CV	3.1	4.9	13.6	4.4	5.8	16.9	3.6	5.9	8.2	15.9	11.7	9.0

ACP: Assay cut point; HPC: High PC; LPC: Low PC; MPC: Medium PC; nAb: Neutralizing antibody; PC: Positive control.

Isotype stability results suggest that all analytes showed 4 h on-bench stability at 2–8°C and ambient temperature as well as acceptable short-term stability (24 h) at 2–8°C (Supplementary Tables 1 & 2).

For assay robustness assessment 1, the incubation time was increased to 90 min, incubation temperature was set to 37°C and shaking speed was increased to 500 r.p.m. For assay robustness assessment 2, incubation time was reduced to 45 min and shaking speed was decreased to 350 r.p.m. The assay selectivity data show that an increase in temperature, shaking speed and incubation times negatively impacted analyte selectivity for IgG, IgM and IgA. Briefly, all 40 naive samples tested positive for IgG (0% selectivity), 35 naive samples tested positive for IgM (12.5% selectivity), 26 naive samples tested positive for IgA (33.3% selectivity) and no naive samples tested positive for nAb (100% selectivity) (Supplementary Table 3). These data indicate that decreased selectivity refers to high levels of false positivity. The decrease in shaking speed and incubation times had less impact on the selectivity assay. No naive samples tested positive (100% selectivity) for IgG, 12 naive samples tested positive for IgM (69.2% selectivity) and one naive sample tested positive for IgA and nAb, respectively (97.4% selectivity). In addition, PC samples did not test positive for nAb in the increased settings robustness assay (0% sensitivity) (data not shown). Robustness data demonstrate the importance of maintaining ambient temperature incubation and accurate incubation times for assay performance.

Cross-reactivity is a concern in the development of all SARS-CoV-2 serology assays [20]. The prevalence of coronaviruses and other seasonal or chronic viruses are highly population specific. Validation of our assay in an African test population with high prevalence of HIV and hepatitis B virus raised concern over assay interference. HIV interferes with the host immune response and may have detrimental consequences for humoral immunity. HIV patients and those with chronic inflammatory conditions such as RA were included in a cross-reactivity panel. Data for IgG, IgM and IgA antibodies were normalized to the HPC mean as previously described and were screened against the set ACPs. The full dataset is represented in Supplementary Table 4. The results of the cross-reactivity assessment are summarized in Table 5. The cross-reactivity assessment suggests that a cross-reactivity (CR) specificity (calculated from the ratio of FPs detected in the panel of 105 subjects), ranged from 85.7–92.4% with IgM having the highest level of false positivity.

**Table 5.** Summary of the number of samples with analytes detected in cross-reactivity panel.

Disease	Analyte combination	Analyte ID			
		IgG	IgM	IgA	nAb
RA (n = 5)	Single analyte	0	0	0	1
Psoriasis (n = 4)	Single analyte	0	0	1	0
	IgG + IgM	1	1	0	0
CIDP (n = 14)	Single analyte	1	1	1	2
	IgG + IgM	1	1	0	0
MG (n = 38)	Single analyte	1	2	1	6
	IgG + IgM	1	1	0	0
	nAb + IgM	0	1	0	1
HBV (n = 20)	Single analyte	1	3	0	1
	IgG + IgA	1	0	1	0
	IgM + IgA	0	2	2	0
HIV (n = 20)	Single analyte	2	2	2	0
	IgM + IgA	0	0	0	0
Syphilis (n = 4)	Single analyte	0	1	0	0
Total analytes detected (n = 105)		9	15	8	11
CR specificity (%)		91.4	85.7	92.4	89.5

CIDP: Chronic inflammatory demyelinating polyneuropathy; CR: Cross-reactivity; HBV: Hepatitis B virus; HIV: Human immunodeficiency virus; MG: Myasthenia gravis; nAb: Neutralizing antibody; RA: Rheumatoid arthritis.

**Figure 2.**
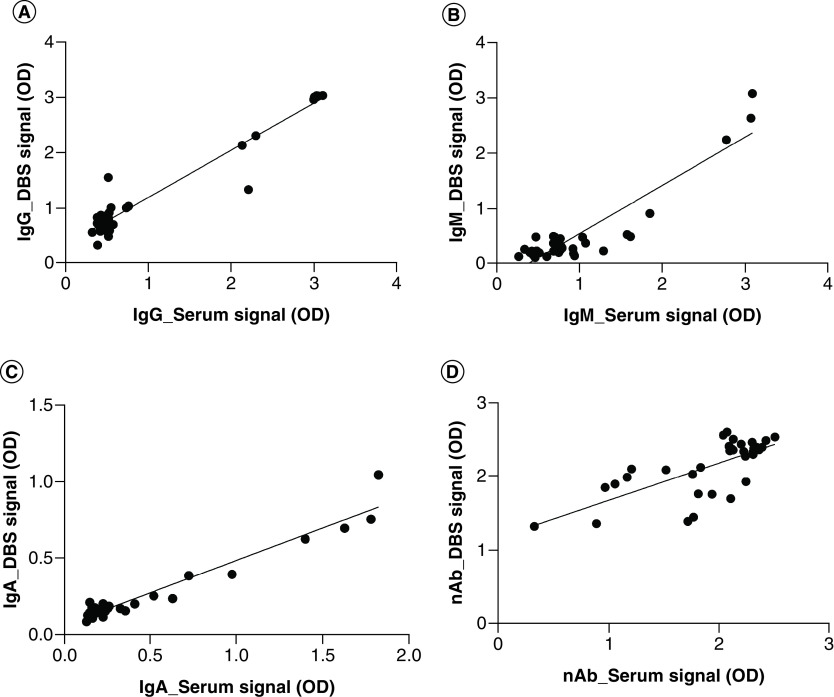
Serum & hemaPEN^®^ dried blood spot signal correlations. Naive and convalescent samples from serum and DBS sample matrices were measured for IgG **(A)**, IgM **(B)**, IgA **(C)** and neutralizing antibody **(D)** analytes. Signals from each matrix were plotted on corresponding axis (n = 34). DBS: Dried blood spot; OD: Optical density.

Another important consideration during the validation of this assay was the application of alternative sample matrices and collection devices. Using serum and DBS convalescent and naive samples collected from the same individuals, the associations between signals generated from each matrix were calculated. Results (summarized in Figure 2) indicate significant positive correlations (p < 0.001) between serum and DBS signals for IgG, IgM, IgA and nAb analytes for all samples (R^2^ = 0.937, R^2^ = 0.839, R^2^ = 0.939 and R^2^ = 0.501, respectively). Additionally, Spearman correlation matrices indicate significant associations (p < 0.005) between serum and DBS final interpreted results for IgG, IgM, IgA and nAb analytes (r = 0.872, r = 0.500, r = 0.781 and r = 0.814, respectively.). These findings are in line with similar studies [23–25]. Although DBS sampling does reduce sensitivity [8,26], our assay consistently demonstrated a similar level of sensitivity when the same samples were analyzed in serum. Care should be taken during sample collection, as sampling differences may affect overall DBS sample quality and introduce variability. The applicability of our assay to remote sample collection becomes particularly important considering vaccine studies and rollout, especially in this new world of social distancing. Although microsampling devices have higher overhead costs, DBS sample collection facilitates rapid, decentralized and stable sample collection as well as providing an easier means of collecting samples from pediatric patients and those who ordinally have difficulty with venous blood collection. In addition, modern devices, such as the hemaPEN^®^ provide more controlled sample volumes and improved consistency by minimizing impact of volumetric and homogenetic effects associated with conventional peripheral blood collection on storage cards.

Vaccines routinely undergo Phase IV clinical after marketing to assess the effects of minor changes to the formulation or vaccine strain, and/or to further optimize parameters that cannot be assessed in the traditional clinical trial setting. Vaccines are typically administered to healthy persons and in large numbers; thus, rapid and continuous benefit–risk monitoring is essential. This is especially true for the COVID-19 vaccine candidates, given their expedited development and rapid global implementation. Adverse drug reactions or vaccine-associated adverse events of special interest are likely to include allergic, inflammatory and immune-mediated reactions.

One of the primary criteria for an effective vaccine is the development of nAbs in the vaccinated individual. nAbs are vital for an effective immune response to infection and principally block the binding of the S1 spike protein to the ACE-2 receptor in host cells [27]. Low nAB titre is detrimental in patients and increases the possibility of reinfection. Studies have indicated that low nAB titres are normally associated with milder symptoms in patients [28–30]. The detection of nABs using serological testing is therefore a clinically relevant inclusion in our panel.

**Figure 3.**
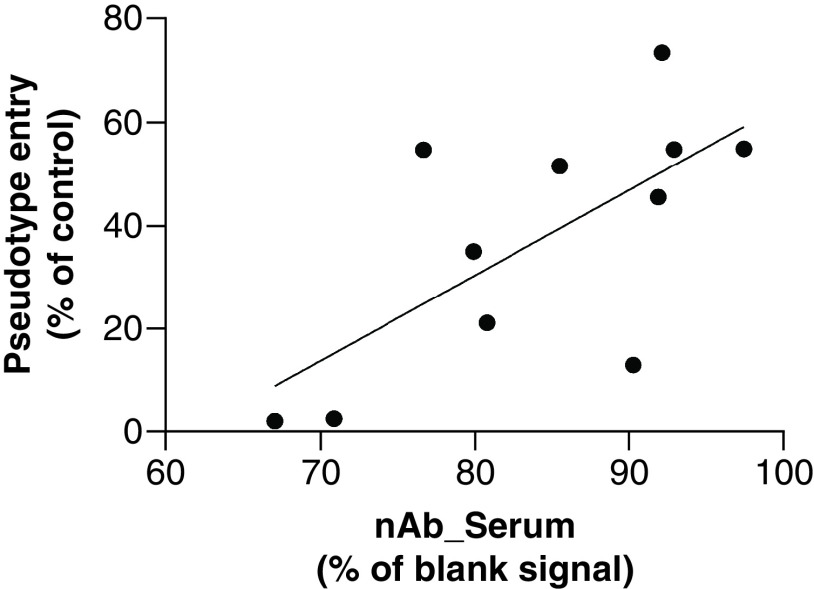
Functional SARS coronavirus-2 pseudotype entry inhibition correlation to serum neutralizing antibody signals. Convalescent samples were analyzed using an *in vitro* functional measurement of SARS coronavirus-2 neutralization and using the solid phase ELISA assay. Pseudotype entry was plotted against blank corrected nAb signal (n = 11). nAb: Neutralizing antibody.

Currently, the gold standard assay for the detection of nABs involves *in vitro* neutralization of live SARS-CoV-2 viral particles [20]. As this assay is time consuming, requires biosafety level-3 (BSL3) level safety and specialized training, a solid-phase assay capable of mimicking *in vitro* neutralization would be of great benefit. An *in vitro* assay quantifying the entry of pseudotyped RVP into 293T-hsACE2 cells following treatment with convalescent donor serum was correlated against the solid phase LBA results attained form the serological assay. Signal data, expressed as percentage of the blank signal, correlated positively with pseudotype entry inhibition at 1:10 dilution, expressed as a percentage of the control (R^2^ = 0.458, p < 0.05), indicating a significant association (p = 0.0098) between *in vitro* flow cytometric data and the ELISA serology assay (Figure 3). This is a critical aspect of the assay and increases its diagnostic relevance. This assay can therefore potentially serve as a surrogate for the traditional *in vitro* approach.

## Conclusion

In this study, we validated a serum-based SARS-CoV-2 serology assay for the detection of IgG, IgM, IgA and nAb antibodies. The assay demonstrated high DSn, DSp, and low cross-reactivity with other viruses and in patients with chronic inflammation. Our solid-phase assay correlated positively with the gold-standard nAb *in vitro* assay as well as with samples collected by remote DBS sample collection – hemaPEN^®^. This assay is applicable for use in clinical trials for infection monitoring, as well as in vaccine trials for monitoring efficacy and long-term postmarketing antibody levels in immunized individuals.

## Future perspective

With the most optimistic of predictions still theorizing the continuation of the global pandemic for the next 2 years, the importance of vaccine rollout, treatment and monitoring has become ever-more crucial. We trust that LBA assays like those described in this article would be valuable tools in evaluating the effectiveness of treatment and social strategies leading to the eventual management of the COVID-19 virus and a successful worldwide vaccination program.

**Summary points**

- Validation of rapid, semiquantitative SARS coronavirus-2 serology assays in serum for detection of IgG, IgM and IgA isotypes and neutralizing antibodies.
- The solid-phase ligand binding assay demonstrates correlation with the neutralization of SARS coronavirus-2 viral entry using equivalent serum samples *in vitro*.
- Strong positive correlation between signals attained for SST serum and hemaPEN^®^ (Trajan Scientific and Medical) dried blood spot matrices.
- The validated assay is applicable to antiviral and vaccine studies, and appropriate postmarketing surveillance, which can include remote sampling collection using hemaPEN^®^ devices.
